# Computational Insights Into the Influence of Substitution Groups on the Inclusion Complexation of β-Cyclodextrin

**DOI:** 10.3389/fchem.2021.668400

**Published:** 2021-05-21

**Authors:** Xianghua Yan, Yue Wang, Tong Meng, Hui Yan

**Affiliations:** ^1^ School of Pharmaceutical Sciences, Liaocheng University, Liaocheng, China; ^2^ School of Chemistry and Chemical Engineering, Liaocheng University, Liaocheng, China

**Keywords:** 2-hydroxyprohyl-cyclodextrin, sulfonated butyl-cyclodextrin, molecular dynamic simulation, inclusion complex, conformational change

## Abstract

Cyclodextrins (CDs) and their derivatives have good prospects in soil remediation application due to their ability to enhance the stability and solubility of low water-soluble compounds by inclusion performance. To investigate the effect of different structural properties of cyclodextrin and its derivatives on the inclusion complexation, molecular dynamic (MD) simulations were performed on the inclusion complexes formed by three kinds of CDs with polycyclic aromatic hydrocarbons (PAHs). Based on neutral β-CD, the other two CDs were modified by introducing substitutional groups, including 2-hydroxypropyl and sulfonated butyl (SBE) functional groups in the ring structure, called HP-CD and SBE-CD. MD results show that PAH can merely enter into the cavity of SBE–β-CD from its wide rim. The substitutional groups significantly affect the structure of CDs, which may also cause the flipping of the glucose units. However, the substitutional groups can also enlarge the volume of the hydrophobic cavity, resulting in a tight combination with the guest molecules.

## Introduction

Polycyclic aromatic hydrocarbons (PAHs) can accumulate in soils, severely harming the environment and human health. Kaur and coworkers found that both the carbamates and organophosphate pesticides form quick and stable complexes with essential metal ions of soil/water medium. Apart from the direct toxicity of pesticides, complexation of free metal ions impoverishes the soils (Kaur et al., 2017). The degradation of three isolated bacterial strains on the glyphosate could be inhibited by Fe(III), Cu(II), and HA (Singh et al., 2019). 3,5,6-Trichlorosalicylic acid (TCSA) does not show good antibacterial activity, but its metal complexes have shown better activity for the selected bacterial strains with good degree of selectivity (Kumar et al., 2017; Kumar et al., 2019). PAHs adsorbed on soils or sediments are very difficult to degrade (Wang and Brusseau, 1995; Brusseau et al., 1997; Reid et al., 2000; Allan et al., 2006; Abdel-Shafy and Mansour, 2016). Soil leaching (McCray and Brusseau, 1998), known as an efficient soil remediation technology, has obtained rapid development in recent years. Through injecting elutriation agents, such as surfactant solution, into the soil, the organic contaminants absorbed on the soil would transfer from the solid phase or nonaqueous phase liquid to the aqueous phase (Guha and Jaffe, 1996; Bueno-Montes, 2011). However, elutriation agents may cause secondary pollution to the soil and environment.

Cyclodextrins (CDs) are a family of cyclic oligosaccharides, consisting of a macrocyclic ring of glucose subunits linked by α-1,4 glycosidic bonds (Loftsson and Brewster, 1996; Dass and Jessup, 2000; Abdel-Shafy and Mansour, 2016). CDs have a unique structure with a hydrophobic interior and hydrophilic exterior. Due to its unique structure, the cavity of the CD can encapsulate other molecules or ions by the inclusion performance, which can solubilize poor water-soluble substances (Eastburn and Tao, 1994). Meanwhile, CDs are also environmentally friendly (almost no secondary pollution) and have a low soil adsorption rate. Besides, water solubility can be improved by the derivatization of native CDs. Because of these advantages, CDs and their derivatives have a good application prospect in the field of soil remediation (Loftsson and Masson, 2001; Jessel et al., 2006; Crini, 2014).

At present, most researches have been focusing on the desorption of hydrophobic organic pollutants in soil by the hydroxypropyl-cyclodextrin (HPCD). For instance, Mayer et al. (2005) found that HPCD can significantly improve the molecular diffusion efficiency of contaminants in the unstirred soil layer. Allan et al. (2007) concluded that in the study of the soil mud system, HPCD increases the rate of microorganism degradation of PAHs and phenolic substances in soil. The extraction of PAHs below the four rings in soil by cyclodextrin (HPCD) solution, which can reflect interactions between PAHs and the soil, can also predict PAHs’ bioavailability in the soil. This provides an easy and fast way to determine the ecological risk in the soil and evaluate the feasibility of biometric repair of the different soils contaminated by persistent organic pollutants (POPs). Cyclodextrins (HPCD) have the potential for the replacement of surfactants to enhance the dissolution and degradation of hard-to-degrade organic pollutants in the soil (Reid et al., 2004). Brewster et al. (2008) found that hydrophilic cyclodextrins might be useful formulation adjuncts in supersaturating drug delivery systems. Maeda et al. (2018) concluded that SBE–β-CD can increase the solubility, stability, and antioxidant activity of S-(-)-equol (SEq) by complexation with SEq more effectively than SEq alone.

Although cyclodextrins and their derivatives have good prospects of soil remediation application, the inclusion performance of different cyclodextrins with the guest molecules shows great differences. Limited by the experimental technique, it is a challenge to explore the microcosmic mechanism behind the different performance of various CDs (Guha and Jaffe, 1996; Bueno-Montes, 2011). The different performance between the CDs should be related to their different structures, especially the substituted groups, such as hydroxypropyl and sulfonated butyl functional groups, that may affect the cavity structures. Besides, both the positions and degrees of substitution may also influence the cavity structure (Erdős et al., 2021). The structural changes, including cavity self-closure and flipping of glucose units, could conversely affect the inclusion capability of the CDs.

To characterize the inclusion abilities of the different kinds of CDs from a microscopic view, theoretical studies, especially molecular dynamics simulations, are certainly appropriate to investigate such problems. To date, the complexation of small-ring cyclodextrins (α-CD, β-CD, and γ-CD) and various small guest molecules have been widely studied theoretically (Yuan et al., 2008; Schönbeck et al., 2010; Concha-Santos et al., 2013; Yuan et al., 2015; Malanga et al., 2016; Nutho et al., 2017; Erdős et al., 2020; Erdős et al., 2021). The theoretical studies have provided detailed information on the inclusion processes and orientation of guest molecules inside the hole. As for the native CD derivatives modified by the substituted functional groups, the theoretical studies on their inclusion are relatively scarce. Recently, Rungrotmongkol and his coworkers studied the effects of different numbers of hydroxypropyl substituents on the conformational changes at different positions of β-CD using replica exchange molecular dynamic simulation (REMD) (Hukushima and Nemoto, 1996; Sugita and Okamoto, 1999; Nutho et al., 2017; Kerdpol et al., 2019).

To explore the different performance of β-CD and its derivatives on contaminant inclusion, we performed classic molecular dynamics simulations to investigate the effect of substitutional groups on the inclusion ability of β-CD. Two types of substitution, including 2-hydroxypropyl (HP) and sulfonated butyl (SBE) functional groups, were investigated. Under the same substitution degree, four HP or SBE groups were connected to the O_2_ atoms on the glucopyranose units. The complexation structures of a PAH compound anthracene (ANT) inside native β-CD and the modified derivatives HP–β-CD and SBE–β-CD were compared. Through analyzing the structural distortion, cavity self-closure of HP–β-CD and SBE–β-CD, and interactions between host and guest molecules, the inclusion ability of native β-CD and its derivatives were explored.

## Computational Methods

**FIGURE 1:**
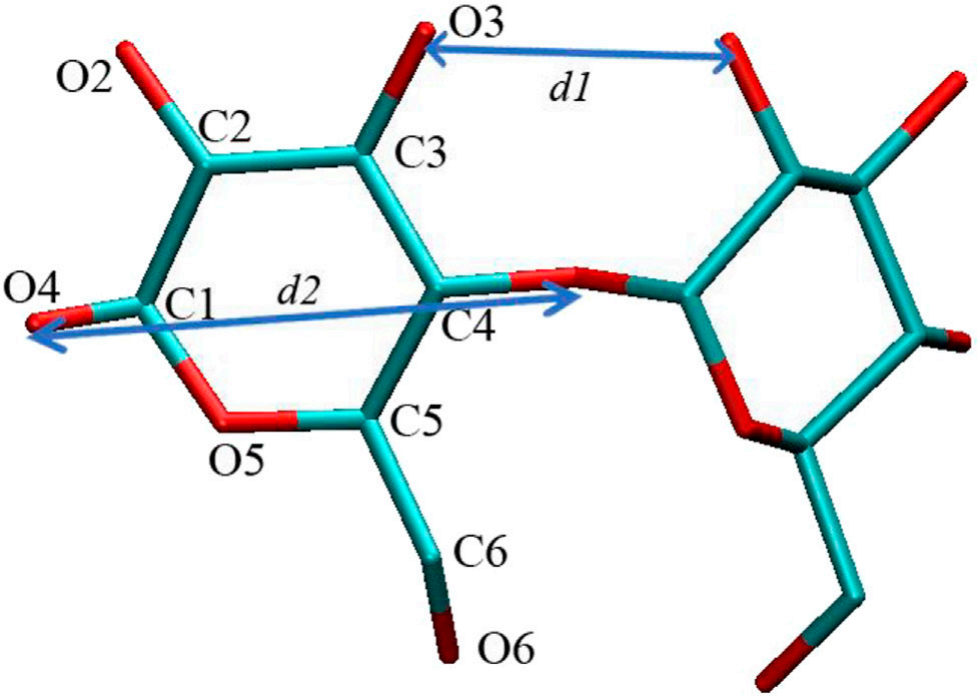
Atomic labels in glucose units of cyclodextrin (CD) ring and the two defined distances.

The structure and force field topology files of the native β-CD were taken from the automated topology builder (ATB) server (Malde et al., 2011; Stroet et al., 2018). The two derivatives HP–β-CD and SBE–β-CD were prepared by connecting four 2-hydroxypropyl (HP) or sulfonated butyl (SBE functional groups to the O2 positions of α-d-glucopyranose units, as shown in Figure 1. The substitution positions were all at the large opening end, the substitution degree was set as four, which is according to the experimental observations. The coordinates of the configurations for the three kinds of CDs were provided in the Supplementary Materials. Then the molecular dynamic simulations were performed to study the inclusion process of the ANT molecule within the three kinds of CDs. The simulated models were built according to the method previously reported in the literature (Snor et al., 2007; Kicuntod et al., 2016). Briefly, the CDs were first placed in a simulation box with the center of mass (COM) located at the center of the coordination system. The two openings on both ends of the CDs were oriented toward the *z*-direction. Two inclusion directions of ANT into the CD cavity were taken into consideration, that is, the two openings of the CD. The guest molecule was placed at the two positions with its long molecular axis coinciding with the central axis of the CDs. Finally, water molecules were filled into the box, and Na^+^ ions were added for the SBE-CD system. All the simulated systems were summarized in Supplementary Table S1 in the Supplementary Materials.

All calculations were performed using the Gromacs 2019 software (Hess et al., 2008; Pronk et al., 2013; Abraham et al., 2015; Páll et al., 2015) with the GROMOS96 54a7 force field (Malde et al., 2011). The united atom force field parameters for all the CDs and the ANT were derived from the automated topology builder (ATB) server (Malde et al., 2011; Stroet et al., 2018). The force field parameters used in this work were all obtained from the ATB server, in which the bonded and van der Waals parameters were taken from the GROMS 54a7 parameter set. For the electrostatic interactions, the atomic charges were calculated using the ESP method of Merz–Kollman based on quantum optimization calculations at the B3LYP/6-31G* level. The obtained topology files, including force field parameters and configurations of the studied components, were provided in the Supplementary Material. Water molecules were described by the simple point charge/extend (SPC/E) potential (Berendsen et al., 1987), which provided a good representation of the structural properties of cyclodextrin systems following the previous works (Khuntawee et al., 2015; Gebhardt et al., 2018). To validate the effect of the different water models on the simulation results, we still performed simulation using SPC and TIP3P models. The results are discussed in the following Results and Discussion section. Molecular dynamic (MD) simulations were performed after the minimization with the steepest descent method. The simulations were performed under the NPT ensemble at 1 atm and 298°K, corresponding to the normal condition. The v-rescale thermostat and Berendsen barostat were used to maintain the system temperature and pressure, respectively (Berendsen et al., 1984; Bussi et al., 2007). The LINCS method was applied to constrain the bond length in the simulation (Hess et al., 1997). The long-range electrostatic interactions were calculated using the particle mesh Ewald (PME) method (Essmann et al., 1995), while the short-range pair interactions were treated using the Lennard Jones potential with a cutoff at 1.2 nm. A timestep of 2 fs was used during the simulations. The MD trajectories were saved every 2 fs and were visualized using the VMD software (Humphrey et al., 1996).

Several statistical methodologies were used to explore the microscopic properties, including the routine analysis of MD simulations, such as structural properties and radial distribution function (RDF). In addition, the structural distortion of glucose units was analyzed in terms of free energy, and the intermolecular interactions between guest and host molecules were analyzed using the independent gradient model (IGM) (Lefebvre et al., 2017; Lefebvre et al., 2018). The specific details are provided in the following Discussion section.

## Results and Discussion

### Inclusion Structures of Anthracene Within Each Cyclodextrin

**FIGURE 2:**
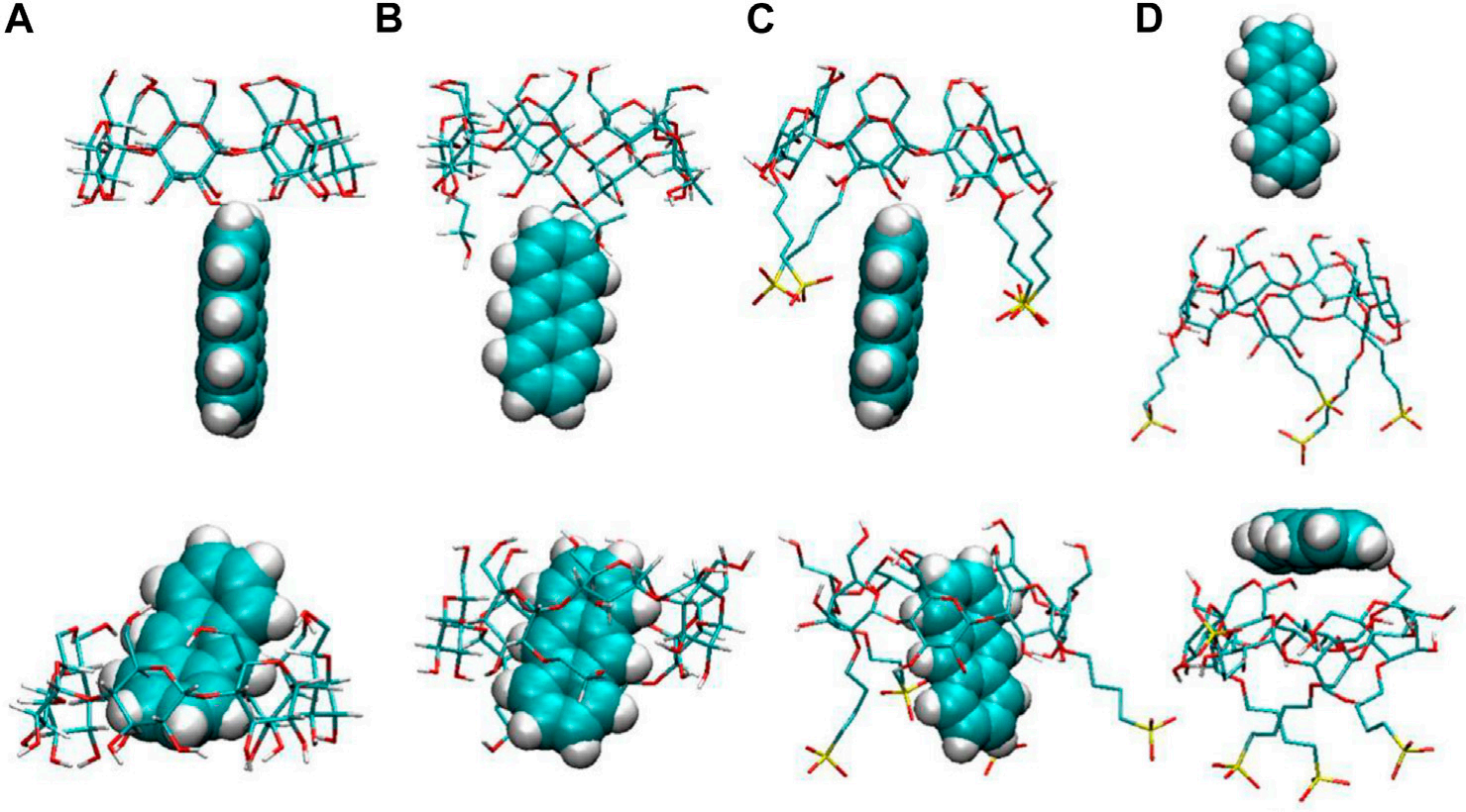
Configurations of anthracene (ANT) with each CD: **(A)** β-CD, **(B)** HP–β-CD, and **(C)** and **(D)** SBE–β-CD. The top and bottom panels show the configurations at the beginning and end of the simulated systems, respectively.

**FIGURE 3:**
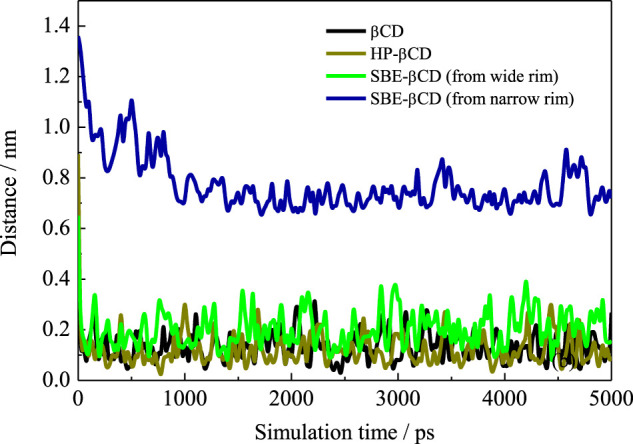
Time evolution of the distance between COMs of ANT and each CD.

We first calculated the system densities to check the equilibrium of the systems. The calculated densities of the systems were provided in Supplementary Figure S1 in the Supplementary Materials. The results showed that the densities of simulated systems were all around 0.992 g/cm^3^, close to bulk water density. Besides, the densities kept steady despite the fluctuations during the simulation. Figure 2 shows the configurations for each inclusion system at the end of the simulation. The configurations of ANT entering into the CD cavity from its narrow rim are shown in Supplementary Figure S1 in the Supplementary Materials. In addition, all the simulations were performed twice to check the repeatability of the systems. The results are shown in Supplementary Figures S2–S4; they show good consistency. Generally, the inclusion complexes of ANT within the three CDs were all obtained. The difference is that the ANT molecule can enter into the cavity from both the wide and narrow rims of the native β-CD and HP–β-CD, while it can only penetrate the cavity of the SBE–β-CD from its large rim, that is, from the rim which was connected to the sulfonated butyl groups. This point can be confirmed by checking the MD trajectories, and the inclusion degree of ANT within each CD was investigated by the COM distance between host and guest molecules, as shown in Figure 3. The fluctuation of distances was within the range of 0.2 nm, which showed that ANT can be tightly included inside the cavity.

### Structural Distortion of Glucose Units in Each β-CD

It is noted that the ANT molecule cannot enter into the cavity of SBE–β-CD from the small rim, as shown in Figures 2, 3. We consider this directly related to the conformational distortion of the glucose units in the SBE–β-CD structure. Thus, the conformational changes of the three kinds of β-CDs were investigated according to previous studies reported by Rungrotmongkol and coworkers (Kerdpol et al., 2019). First, the distance $d_{1\left(i\right)}$between adjacent oxygen atoms in two adjacent glucopyranose units was measured to monitor the intramolecular hydrogen bonds of the wide rim. Then, the distance $d_{2\left(i\right)}$ between the same glycosidic oxygen atoms was calculated to monitor the ellipticity of each kind of CDs. The above two distances were labeled onto the structure of β-CD to give a clear illustration, as shown in Figure 1. The probability distributions of the two distances were calculated using the following equation in terms of free energy (Kerdpol et al., 2019):

$$F\left(x,y\right)=-k_{B}T\log[P\left(x,y\right)],$$ (1)

where $k_{B}$ and T are the Boltzmann constant and absolute temperature, respectively. P(x,y) is the probability of the above two distances.

**FIGURE 4:**
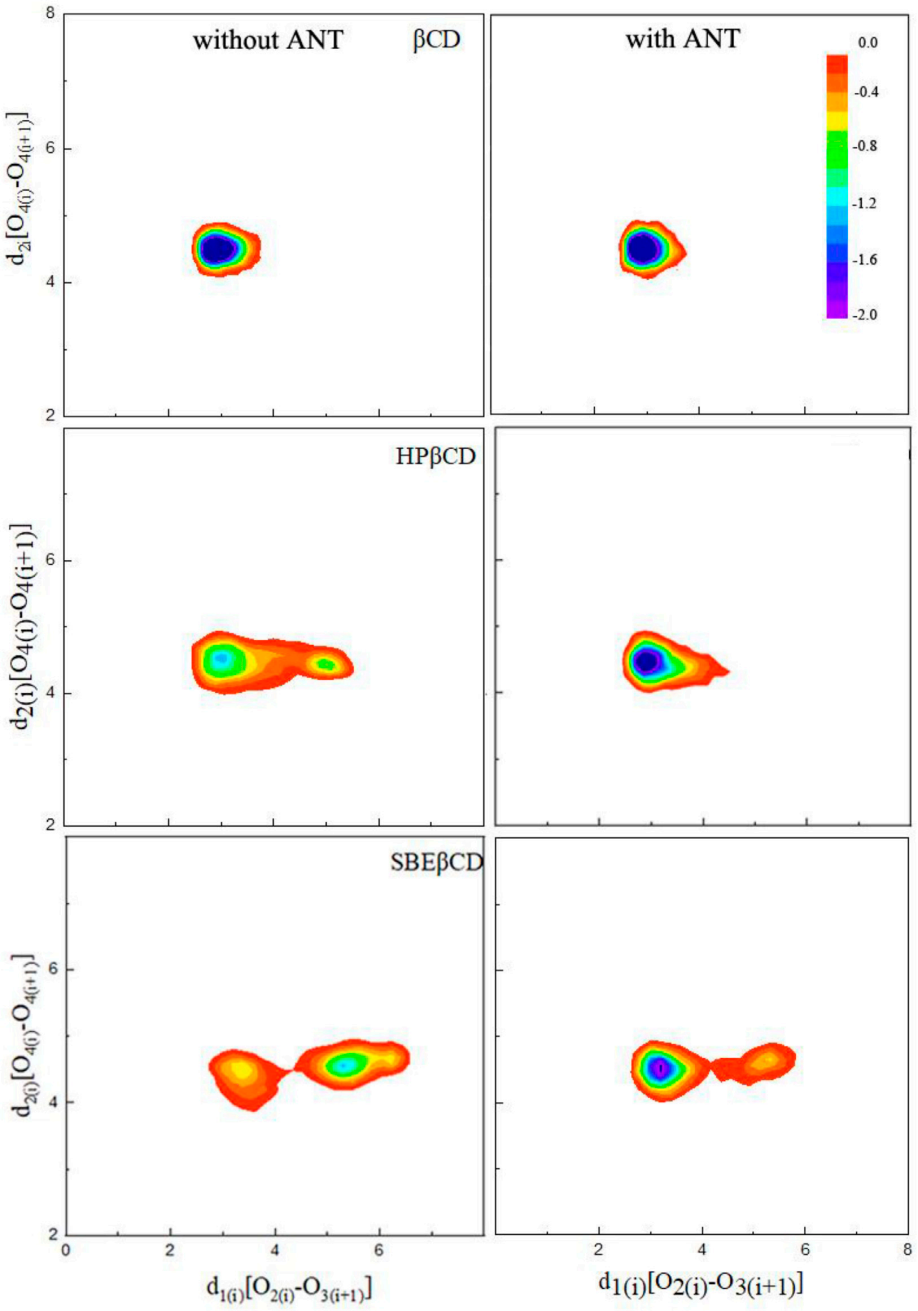
Probability distributions of the two defined distances $d_{1\left(i\right)}$ and $d_{2\left(i\right)}$ in terms of free energy.

For the native β-CD, the conformational minimum was detected with the $d_{1\left(i\right)}$ and $d_{2\left(i\right)}$ distances at about 2.9 and 4.5 Å, respectively, indicating the conical structure of β-CD ring with two ends. When the ANT molecule was included by β-CD, the distribution of the conformational minimum was almost unchanged, as shown in Figure 4. In the cases of HP–β-CD and SBE–β-CD, the contour graphs of the probability distributions both showed two regions of conformational minima, as plotted in Figure 4. This indicates that the structures of native β-CD would be changed due to the substitution of HP or SBE groups. It is noticeable that the probability distribution of $d_{2\left(i\right)}$ distances changed slightly, while the distribution of $d_{1\left(i\right)}$ fluctuated in a wide range of 3.5–6.0 Å. This implied that the conical structure of the CD ring was changed by narrowing down the secondary rim. In particular, the minimum energy conformation of SBE–β-CD mainly existed with $d_{1\left(i\right)}$ distances at about 5.8 Å, which indicated that the macrocyclic ring structure of CD deformed to a greater extent when the SBE substitution groups existed.

When the ANT molecule was packaged inside the cavities of HP–β-CD and SBE–β-CD, it was observed that the cavity structure was returned to its original form. As shown in Figure 4, the probability distributions of the lowest minimum energy conformations were once again concentrated at 3.0 and 4.5 Å for $d_{1\left(i\right)}$ and $d_{2\left(i\right)}$, respectively. For SBE–β-CD, the distribution of $d_{1\left(i\right)}$ still fluctuated in a wide range of 3.5–6.0 Å, suggesting that the SBE substitute groups have a bigger impact on the CD ring structure. Even the ANT molecule was located inside the cavity; the conical structure also was narrowed down. Therefore, it is possible that the binding between ANT and SBE–β-CD would be much tighter than that of the other two complexes.

### Arrangement of the Substitution Groups

**FIGURE 5:**
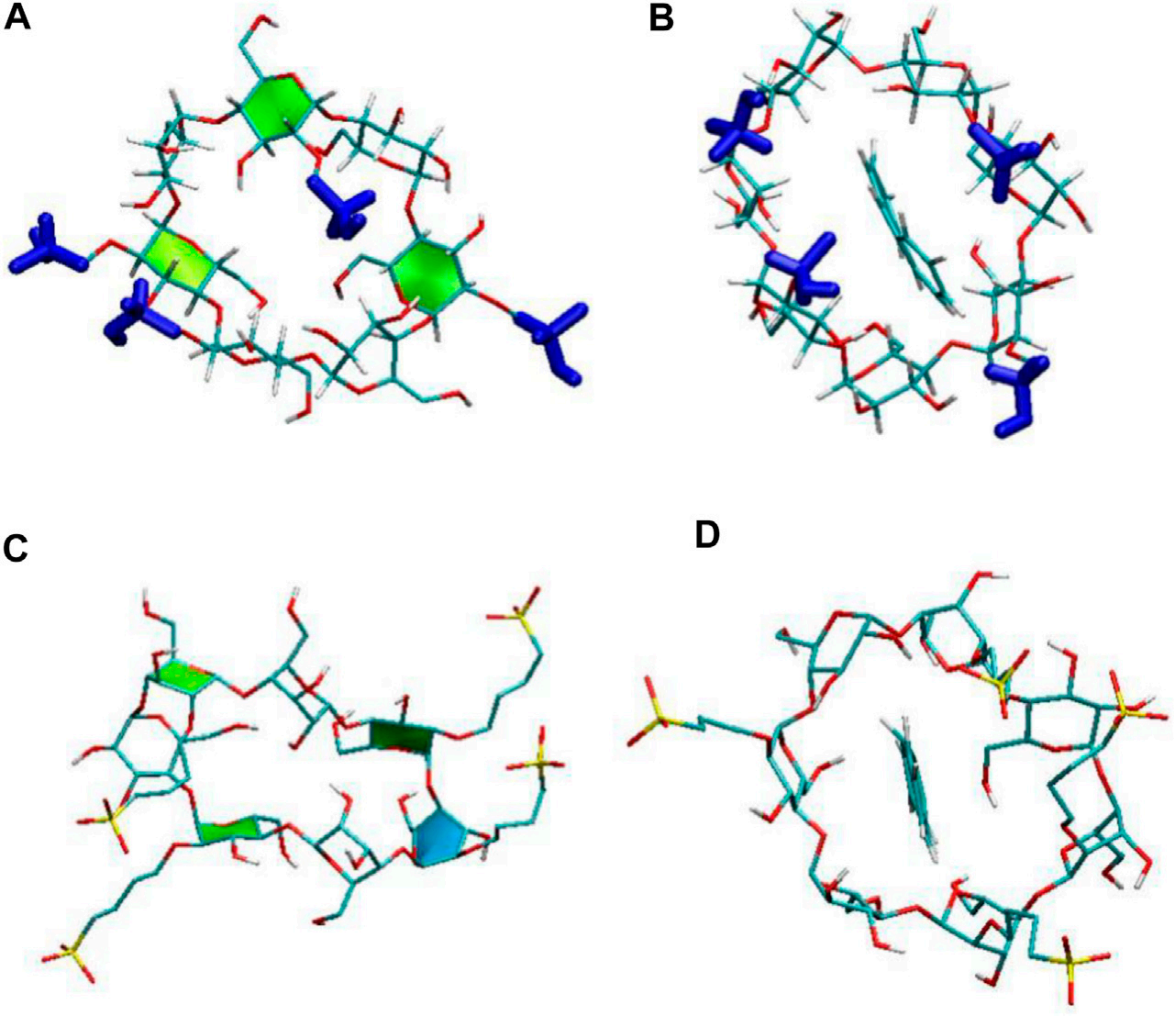
The flipped conformations of HP-CD with **(A)** and without ANT **(C)**. The flipped conformations of SBE-CD with **(B)** and without ANT **(D)**.

The substitutional groups on the ring structure can affect the CD conformations as discussed above. The substitutions could also influence the inclusion performance, because these flexible groups may cause the self-closure of the cavity. Figure 5 shows the configurations of two CDs at the end of the simulation without and with ANT, respectively. It can be seen that these substitutional groups can point toward the cavity interior or exterior due to their flexibility. Meanwhile, the fluctuation of these groups caused the flipping of the glucose subunits, as the highlighted glucose ring shown in Figure 5. The flipping of glucose rings then influenced the distortion of the cyclic ring. The observations of glucose subunits are similar to those of an earlier study reported by Kerdpol et al. (2019).

**FIGURE 6:**
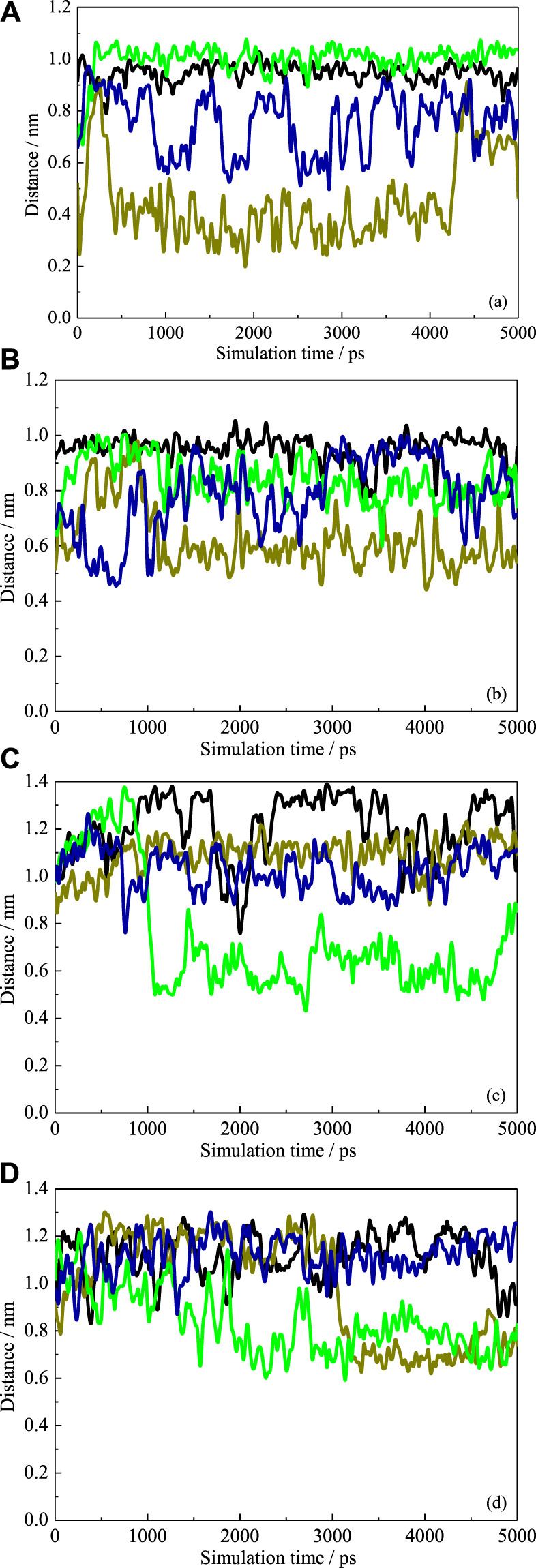
The COM distances between HP-βCD and each substitution group with **(A)** and without ANT **(B)**. The COM distances between SBE-β CD and each substitution group with **(C)** and without ANT **(D)**.

When the substitutional group pointed toward the interior cavity, which would block the CD cavity and then prevent the guest molecule from inserting into the cavity, this may further affect the inclusion performance of each CD. To investigate the arrangement of the substitution groups in detail, the COM distances between β-CD and each substitution group, that is, HP and SBE, were calculated *versus*. simulation time, as shown in Figure 6. In the absence of ANT, it is found that one HP group pointed toward the interior cavity during 80% of the MD simulation period. As shown in Figure 6A, the distance between one HP chain and β-CD COM fluctuated to around 4.0 Å. The configuration (5 ns) shown in Figure 5A exactly shows an HP group pointing toward the interior, leading to the self-closure of the cavity.

**FIGURE 7:**
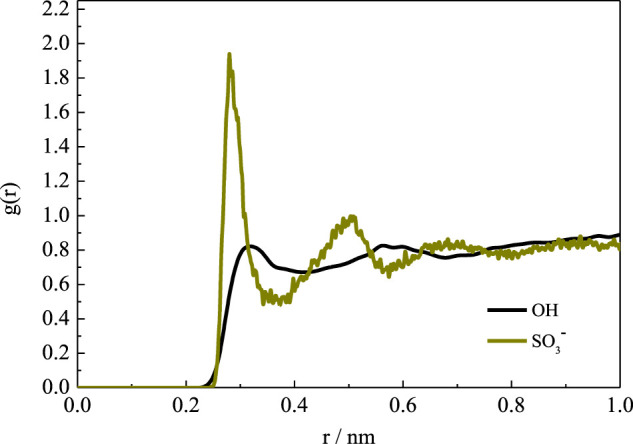
RDFs of water molecules around hydroxy and sulfonate groups.

For the SBE–β-CD, all the distances were more than 5.0 Å during the simulation, suggesting that the four substitutional SBE groups were always pointing toward the exterior of the cavity. This indicates that the SBE groups did not close the wide rim of the CD. On the one hand, the length of SBE groups is longer than that of HP. Therefore, as mentioned above, the flexibility of the molecular chain is greater than that of the HP group, which leads to a greater degree of deformation. On the other hand, the hydration affinity of the sulfonate group was stronger than that of the hydroxy group. This was confirmed by the radial distribution functions (RDFs) of water molecules around the two kinds of groups, as shown in Figure 7. It can be seen that the peak intensity of the first hydration shell of SBE groups was obviously stronger than that of HP groups. Since the CD cavity is hydrophobic, the SBE groups with stronger hydrophilic character prefer to point toward the exterior.

In addition, the RDF results obtained from the simulations with TIP3P and SPC water model are shown in Supplementary Figure S5 in Supplementary Materials. It can be seen that the results show good consistency, except for the small difference in peak intensities.

### Structure of the Inclusion Complex

When the inclusion complex formed, the cavity conformation of each CD has recovered to a great extent, as discussed above. As the distance profiles (Figures 6C,D) showed, almost all the distances between substitution groups and CD COM were more than 5.0 and 7.0 Å for HP-CD and SBE-CD, respectively. This indicated that no substitutional groups extended to the interior of the cavity. It is because of the steric hindrance from the ANT molecule inside the cavity. Meanwhile, we found that all the max distances between substitutional groups and CD COM were decreased when the guest molecule was inside the cavity. For instance, Figure 6C shows that the maximum fluctuation exceeds 1.3 Å in the absence of ANT. While in the presence of ANT, the maximum distances fluctuated around 1.2 Å, the distribution of distance became more concentrated (i.e., 0.5–1.4 and 0.7–1.2 Å in Figures 6C,D, respectively). This indicated that the substitution groups preferred to extend along the axis direction of the cavity and interacted with the guest molecule.

**FIGURE 8:**
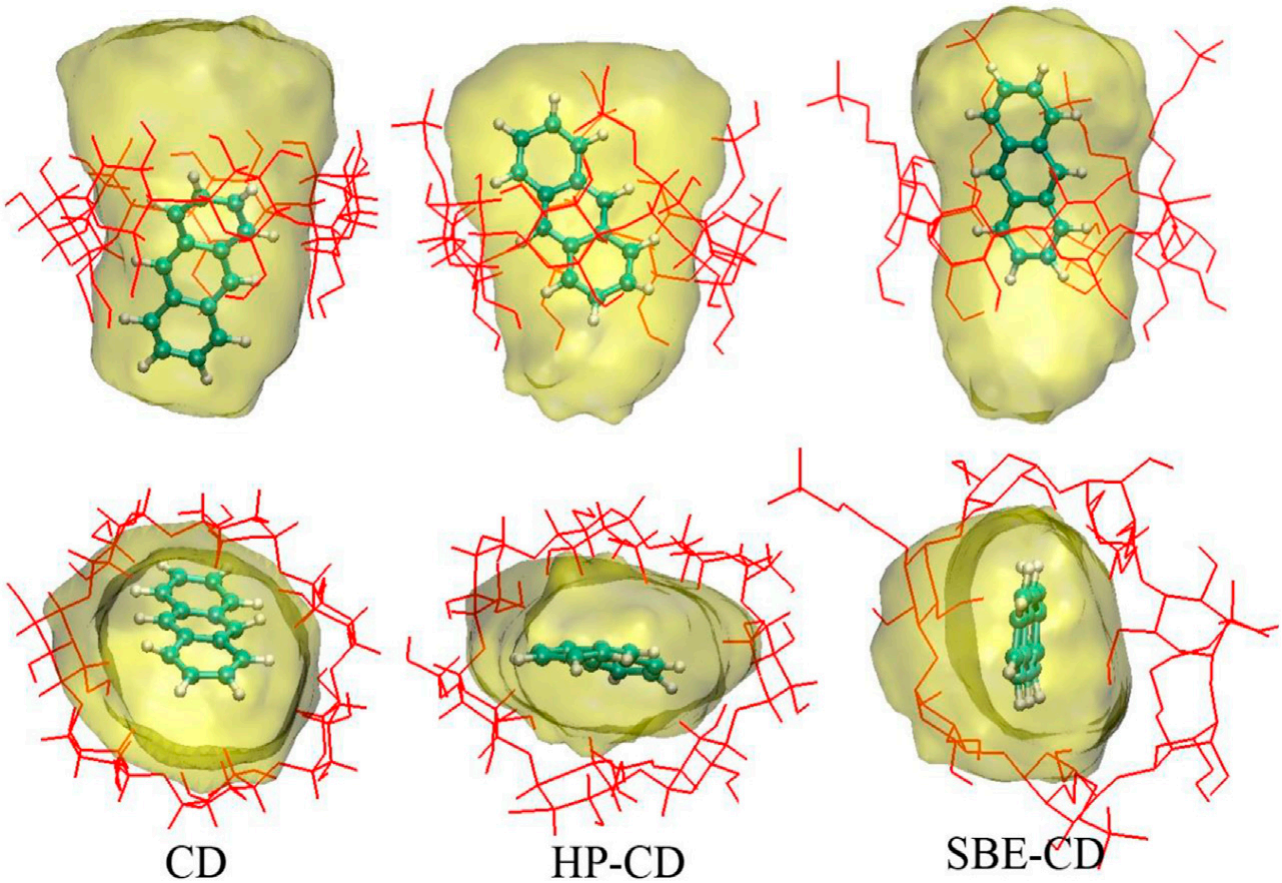
Distribution area of ANT inside the cavity. The top and bottom panels show the axis and radial direction of CD ring, respectively.

To further investigate the inclusion structure in detail, the distribution area of ANT inside the cavity was plotted by superposing the configurations of ANT occurring during the MD trajectories. During this process, the heavy atoms of each CD were restrained, and ANT was allowed free movement. Then, 1 ns MD simulation was performed for each inclusion complex. The composite configurations of ANT inside each CD cavity were shown in Figure 8 by the display mode of molecular surface. The movement area of ANT inside the cavity was divided into two parts. One is along the radial direction of the CD ring, and the other is along the axis direction of the cavity, as shown in Figure 8. In the absence of any substitution groups, it can be seen that the cavity of natural CD kept its elliptical shape during the simulation. Thus, the range of ANT motion in the radial direction of the CD cavity was quite wide. However, along the axis direction, it can be seen that the location area extended beyond the CD rims. This is because of the molecular motion of the long and narrow-shaped ANT molecular along the axis direction.

In the case of HP-CD and SBE-CD, the molecular motion along the axis direction of CD has not changed. The movement trajectories of HP-CD and SBE-CD still exceeded the two cavity rims. However, compared with the inclusion configuration, the movement of ANT near the narrow rim of CD has a little change. However, at the wide rim that connects to the substitution groups, the outmost region of ANT movement was near the margin of the substitutional groups. As discussed above, the substitution groups preferred to extend along the axis direction of the CD cavity when ANT molecule was included. Thus, the substitutional groups enlarged the volume of the inner hydrophobic area inside the cavity, which is helpful to the inclusion of ANT.

In the radial direction, the movement of ANT seems to be greatly hindered in the case of HP-CD and SBE-CD, due to the conformation changes of the CD structure. Figure 8 shows that the molecular motion of ANT was restrained in a narrow and flat region inside the cavity. As discussed above, the cavity contracted inward when ANT was included inside HP-CD or SBE-CD. The cavity contraction was associated with the intermolecular interactions between guest and host molecules on the one hand. But what is more important is the fluctuation of substitutional groups. It can be seen that the conformation of natural CD did not change much when ANT was included inside.

### Interactions Between Guest and Host Molecules

To further investigate the inclusion performance of each CD on ANT, the intermolecular interactions between guest and host molecules were analyzed using the independent gradient model (IGM) (Lefebvre et al., 2017; Lefebvre et al., 2018). Following the developer’s tutorial (Lu, 2020), the IGM analysis was performed based on the final configurations of each complex using Multiwfn software (Lu and Chen, 2012). The IGM function $\delta g^{\mathrm{inter}}$ describing the intermolecular interactions was plotted by mapping the real space function $sign\left(\lambda_{2}\right)\rho \;$on the $\delta g^{\mathrm{inter}}$ isosurfaces with colors. In the $sign\left(\lambda_{2}\right)\rho$ function, $\lambda_{2}$ is the second largest eigenvalue of Hessian matrix of electron density ρ. Besides, to quantificationally explore the contribution of atom pair to the intermolecular interactions, the atoms were colored by mapping the atom pair δg indices on the molecular structure. The atom pair δg index was defined by the developer of Multiwfn (Lu, 2020), which can reflect the contribution of an atomic pair to $\delta g^{\mathrm{inter}}$. The IGM isosurfaces are visualized using VMD software (Humphrey et al., 1996).

**FIGURE 9:**
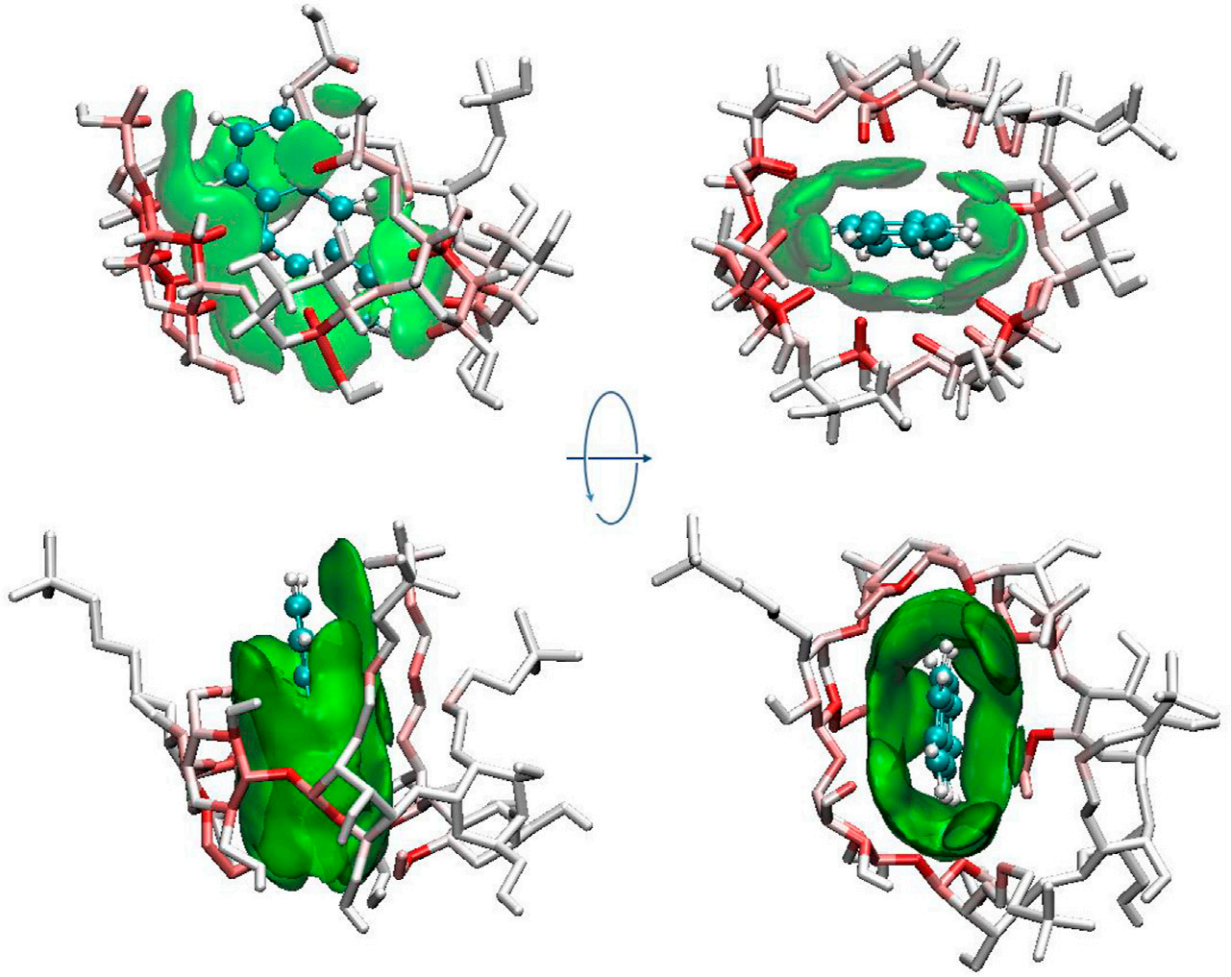
IGM isosurfaces and atom δg indices.

The green isosurfaces between host and guest molecules shown in Figure 9 indicate that the major interaction between ANT and each CD was van der Waals interactions. The van der Waals interaction region mainly located in the CD cavity, suggesting the strong interactions of ANT with its surrounding glucose units. It was also noticed that the substitutional groups can interact with ANT through van der Waals interactions. As shown by arrows in Figure 9, the green isosurfaces occurred between ANT and HP or SBE groups. These interaction regions either grew together with the interaction region that occurred inside the CD cavity or occurred individually. However, the interactions from the substitution groups will surely promote the inclusion effect.

In Figure 9, the atoms of CD were colored by the atom δg indices. The deeper the red color, the larger the atom δg index. The atoms belonging either to CD glucose unit or substitution groups close to guest ANT contributed to the intermolecular interactions, while the interactions from atoms colored in white can be ignored. It can be seen that atoms interacting with ANT distributed around the cavity in HP-CD. While in SBE-CD, a relevant portion of fragments in the CD cavity are not involved in interaction with ANT due to the conformational change of CD structure in the presence of SBE groups. The shrink of the cavity may result in tight inclusion of the guest molecule.

## Conclusion

Molecular dynamic (MD) simulations have been performed to investigate the inclusion behavior of ANT into three kinds of CDs, that is, β-CD, HP–β-CD, and SBE–β-CD. MD results show that ANT can spontaneously enter into the cavity of the three kinds of CD. The difference is that ANT can only enter into the cavity of SBE–β-CD from its wide rim. The effects of the substitutional groups 2-hydroxypropyl (HP) and sulfonated butyl (SBE) on the CD structure were then investigated by analyzing the structural distortion of glucose units of each CD. It is found that the neutral β-CD shows a conical structure, while the existence of HP or SBE groups influenced the structure greatly. The secondary rim would narrow down due to the flipping of the glucose units caused by the fluctuation of substitution groups. When ANT was included inside the cavity, the cavity conformation of the three CDs has recovered to a certain degree. Meanwhile, the substitutional groups extend along the cavity axis and interact with the guest molecule. By analyzing the interactions between each CD and ANT, it is found that the substitution groups enlarged the volume of the hydrophobic cavity. Both the substitutional group and the glucose units would contribute to the interactions with the guest molecule. The conformational change of the CD ring results in the shrinkage of the cavity, which may cause tight binding with the ANT molecule.

## Data Availability

The original contributions presented in the study are included in the article/Supplementary Material, further inquiries can be directed to the corresponding author.
