# Supplementary material for: Computational Insights Into the Influence of Substitution Groups on the Inclusion Complexation of β-Cyclodextrin
Source: Front Chem. 2021 May 21;9:668400. doi: 10.3389/fchem.2021.668400 (PMC8176092; doi:10.3389/fchem.2021.668400)
Supplement: Supplementary file 2 [file DataSheet1.docx]

Supplementary Information for

Computational insights into the influence of substitution groups on the inclusion complexation of β-Cyclodextrin

Xianghua Yan^1,2^, Yue Wang^1^, Tong Meng^1^, Hui Yan^1,^*

^1^School of Pharmaceutical Sciences, Liaocheng University, Liaocheng, Shandong, 252059, China

^2^School of Chemistry and Chemical Engineering, Liaocheng University, Liaocheng, Shandong, 252059, China

*** Correspondence:**Corresponding Author
yanhui@lcu.edu.cn

# **Table S1.** Summary of the Simulated Systems

| Systems | ANT | CD | Water | Na^+^ |
| --- | --- | --- | --- | --- |
| β-CD | 1 | 1 | 17072 | - |
| HP-βCD | 1 | 1 | 17059 | - |
| SBE-βCD | 1 | 1 | 17042 | 4 |

**Figure S1.** Time evolution of the calculated densities for each system.


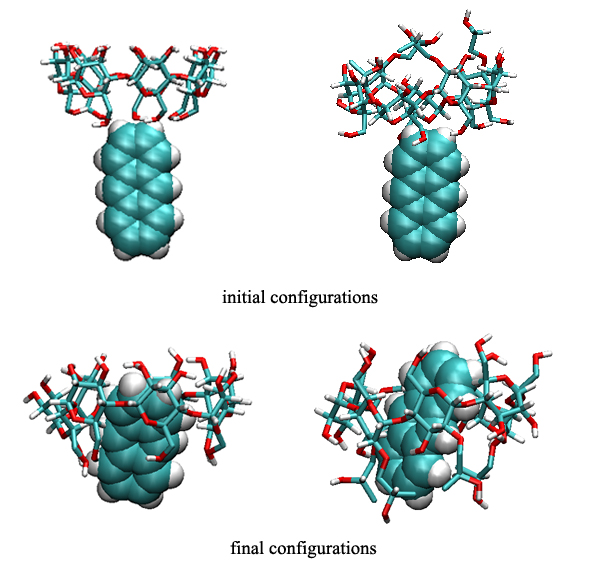


**Figure S2.** Configurations of ANT with βCD and HP-βCD. The ANT molecule was firstly placed near the narrow rim of CD ring.


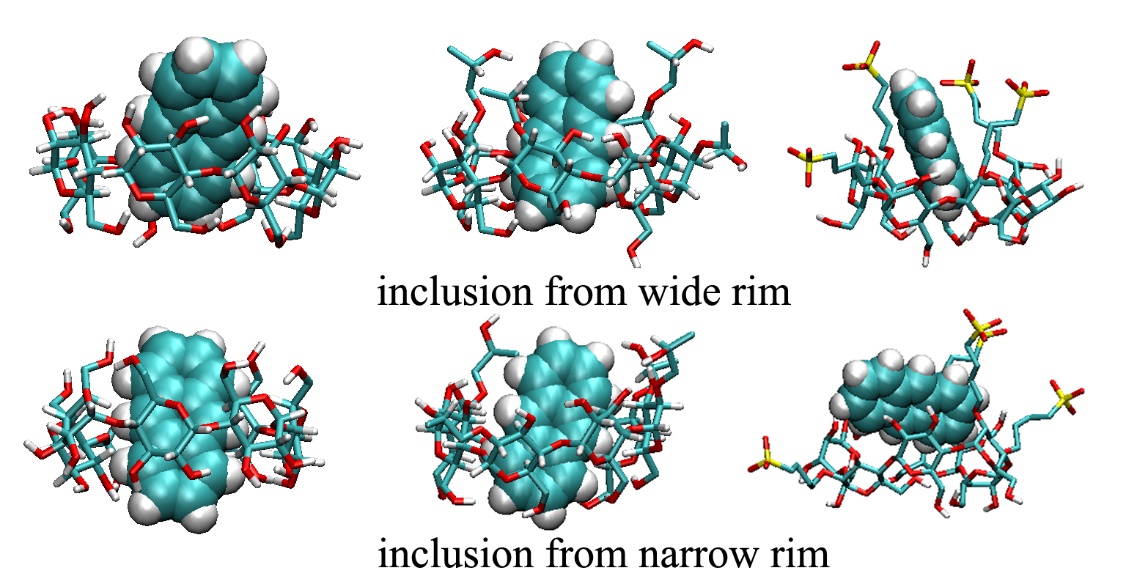


**Figure S3.** The results from the repeated simulations.


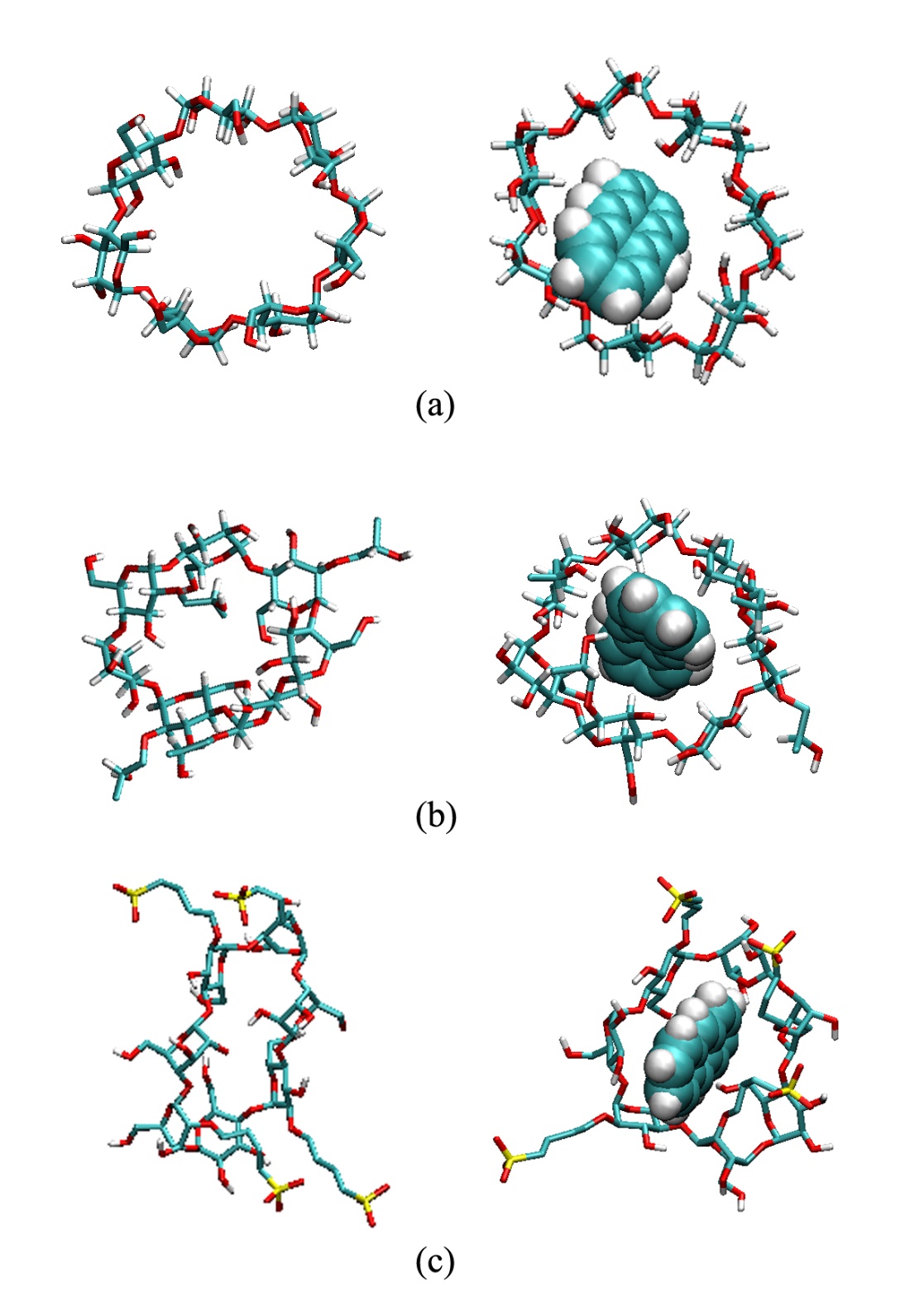


**Figure S4.** The flipped conformations of each CD with and without ANT. (a) βCD, (b) HP-βCD, and (c) SBE-βCD. These results were obtained from the repeated simulations.

Figure S5. RDFs of water molecules around sulfonate groups of SBE-βCD using different water models.
